# Supplementary material for: Different Dynamic Nodal Properties Contribute to Cognitive Impairment in Patients with White Matter Hyperintensities
Source: Brain Sci. 2022 Nov 10;12(11):1527. doi: 10.3390/brainsci12111527 (PMC9688268; doi:10.3390/brainsci12111527)
Supplement: Supplementary file 1 [file brainsci-12-01527-s001.zip › brainsci-1931868-supplementary.pdf]

Supplementary Materials: Different Dynamic Nodal Properties Contribute to Cognitive Impairment in Patients with White Matter Hyperintensities

Table S1. Neuropsychological tests between WMHs

	Mild WMHs (*n* = 39)	Moderate WMHs (*n* = 37)	Severe WMHs (*n* = 28)	F	*p*value
MoCA	21.16 ± 4.32	21.06 ± 3.79	17.81 ± 4.91	5.768	0.04 *
AVLT-study	7.63 ± 2.24	7.31 ± 1.52	6.55 ± 2.00	2.306	0.105
AVLT-immediate	8.17 ± 3.84	7.88 ± 2.65	6.17 ± 3.27	2.878	0.061
AVLT-delay	7.95 ± 3.76	7.31 ± 2.67	6.50 ± 3.13	1.436	0.243
AVLT-recognition	13.53 ± 3.68	13.16 ± 1.87	11.13 ± 3.62	5.361	0.006 **
TMT-A	80.72 ± 33.12	79.12 ± 33.73	105.82 ± 37.72	29.965	<0.001 ***
TMT-B	155.35 ± 70.69	158.16 ± 73.16	207.55 ± 81.24	112.333	0.001 **
TMT(B-A)	68.68 ± 51.19	78.57 ± 50.56	102.24 ± 57.58	3.074	0.030 *
BNT	12.89 ± 1.54	13.25 ± 1.68	12.74 ± 1.40	0.044	0.957

Data are mean ± SD. Abbreviations: HC, healthy control; WMHs, white matter hyperintensities; SD, standard deviation; MoCA, Montreal Cognitive Assessment; AVLT, Chinese Auditory Learning Test; TMT, Trial Making Test; BNT, Boston Naming Test. Volumes are in cubic millimeters. *** significant at 0.001 level, ** significant at 0.01 level and * significant at 0.05 level (2-tailed).

Table S2. Neuropsychological tests between between the mild WMHs group,moderate WMHs group and severe WMHs group

	Mild WMHs: Moderate WMHs		Moderate WMHs: Severe WMHs		Moderate WMHs: Severe WMHs	
	t	*P* value	*t*	*P* value	*t*	*P* value
MoCA	0.106	9.545	3.069	0.003 **	2.954	0.004 **
AVLT-study	0.674	0.502	2.129	0.036 *	1.470	0.145
AVLT-immediate	0.361	0.719	2.291	0.024 *	1.926	0.057
AVLT-delay	0.805	0.423	1.692	0.094	0.923	0.359
AVLT-recognition	−0.209	0.835	2.853	0.005 **	2.973	0.004 **
TMT-A	−5.000	<0.001 ***	−7.470	<0.001 ***	−2.942	0.004 **
TMT-B	13.080	<0.001 ***	12.383	<0.001 ***	0.009	0.993
TMT(B-A)	−0.803	0.424	−2.431	0.017 *	−1.663	0.098
BNT	0.088	0.930	0.291	0.771	0.203	0.840

Abbreviations: WMHs, white matter hyperintensities; MoCA, Montreal Cognitive Assessment; AVLT, Chinese Auditory Learning Test; TMT, Trial Making Test; BNT, Boston Naming Test;Volumes are in cubic millimeters. *** significant at 0.001 level, ** significant at 0.01 level and * significant at 0.05 level (2-tailed).

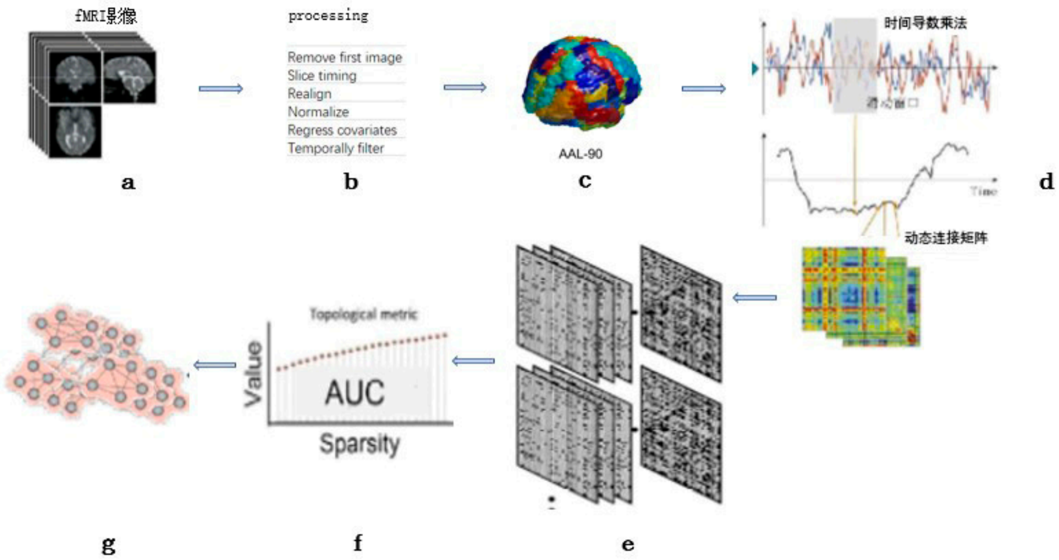

Figure S1. (a) Resting-state fMRI data; (b)preprocessing; (c) the brain was parcellated into 90 distinct regions according AAL(the automated anatomical labelling template), which served as nodes of the individual brain networks;(d)The time course from each region of interest after preprocessing was computed as the averaged BOLD signals within the region. A sliding window comprising 50 TR (120 sec) with a step-gap of 5 TR (12 sec) was applied to regional mean time courses, and for each window we estimated the functional connectivity between pairs of regions using Person correlation;(e,f) individual obtained temporal FC matrix was converted to binary format with a sparsity threshold of 0.05 ~ 0.5 instead of selecting a certain threshold;(g) apply the graph theory to conduct topological properties.[1,2]

Topological properties meanings[3]:

The clustering coefficient reflects the local connectedness of the graph and is the ratio between the number of connections between the direct neighbours of node and the maximum number of possible connections between the neighbours of node Overall, it examines the local interconnection ability of the network.

A small world network is characterised by a high value of local and global efficiency and a small characteristic path length. A small world network may be termed a densely connected local clustered network having a slight characteristic path length that permits a prompt communication between the nodes in the network.

The local efficiency reveals how much the network is fault-tolerant, and demonstrates the efficiency of communication among the first neighbors of node when it is removed.

Degree centrality defined as the number of links (edges) connected to the node.

betweenness centrality represents the fraction of all the shortest paths that pass through a respective node. Nodes with high values of BC can be interpreted as hub nodes that integrate divergent parts of the network

[1] Sun Y, Collinson SL, Suckling J, Sim K (2019) Dynamic Reorganization of Functional Connectivity Reveals Abnormal Temporal Efficiency in Schizophrenia. *Schizophr Bull* **45**, 659-669.

[2] Luo L, Li Q, You W, Wang Y, Tang W, Li B, Yang Y, Sweeney JA, Li F, Gong Q (2021) Altered brain functional network dynamics in obsessive-compulsive disorder. *Hum Brain Mapp* **42**, 2061-2076.

[3] Rubinov M, Sporns O (2010) Complex network measures of brain connectivity: uses and interpretations. *Neuroimage* **52**, 1059-1069.
